# Report of the Commissioners Appointed to Take the Census for Ireland for 1841

**Published:** 1844-07-01

**Authors:** 


### Report of the Commissioners appointed to take the Census for Ireland for 1841. (With Classified Tables of Deaths, and Report thereon). By *William Robert Wilde*, Surgeon. Dublin, 1843.

Any surprise we may have felt at the publication of the Irish Census being delayed so much beyond the appearance of that of Great Britain, was completely removed upon the reception of this portly tome; and upon examination of its contents and passing the eye over its seven or eight hundred folio pages of minutely elaborated tables, we only wonder that any diligence or perseverance could have prepared it so soon, especially when the additional difficulty the imperfect character of some of their materials entailed upon the Commissioners is considered.

The important benefits conferred upon England by the Registration Act, are now generally acknowledged, and will become more and more apparent as the accumulated figures give a larger field for examination and inference; and it may well be inquired, why Scotland and Ireland, (that is eleven out of the twenty-seven million of our population,) should be excluded from benefits which, although now only realising, were well known as inevitable prior to the passing of the act. While this exclusion continues, the demonstration of the condition of this country, as a whole, is of course impracticable, much to the disappointment and, sometimes, to the deception, of foreign staticians; and the comparison of it parts, so essential to its welfare and improvement, must be imperfect.

The present Census is a laudable attempt to remedy some of the evils the great neglect of statistical inquiries in Ireland has given rise to; and to place before the public information respecting some of the points as regards Ireland, which the Registrar's Reports have so beneficially diffused concerning England. The Commissioners were well aware of the importance of their task, but also found it attended with some considerable difficulties. Alluding to the various collateral subjects into which they had endeavoured to inquire,—they say—

"Many inquiries of a similar nature might, no doubt, have been pursued with advantage to a correct, knowledge of the condition of the people. We felt, in fact, that a Census ought to be a Social Survey, not a bare Enumeration. But we were restrained by the apprehension that jealousy and prejudice might be excited, if we made our inquiries too searching and too minute. People are slow to see that questions relating to themselves and their households can have any bearing upon the general good, and forget that, in accounts of large numbers, the individual is wholly lost sight of in the average, but that the average can only be obtained by an accurate knowledge of all that pertains to the individual."

If the Registrar-General has been fortunate in meeting with such an able coadjutor as Mr. Farr, the Irish Census Commissioners have been no less so in securing the services of Surgeon Wilde. His elaborate analysis of the deaths which occurred during the ten years preceding the taking the Census is a masterly performance, and his Report a most interesting document, as exhibiting the defective condition, or rather the entire absence, of registration of death in Ireland, for even Bills of Mortality seem scarcely to have existed there, a candid exposition of the amount of information derivable from this *ex post facto* registration by means of the Census, and as opening new ground for, and smoothing many of the difficulties of the subject to future inquirers.

To this part of the publication we intend chiefly to confine ourselves; but may just state, previously, some of the general results arrived at by the Commissioners.

In the first place, we have to remark the small increase of population which has taken place between 1831-41, compared to 1821-31: the numbers being, for the year 1821, 6,801,827; for 1831, 7,767,401; *i. e.* an increase of about $14\frac{1}{4}$ per cent.; and for 1841, 8,175,124, or an increase of only $5\frac{1}{4}$ per cent. There is some season to believe that the Census for 1821 was rather under, and that for 1831 rather over-stated. Emigration has been the principal cause of the diminution, for Ireland being solely an agricultural country, has not the means of employing an increasing population. In *Scotland,* the population increased $10\frac{8}{10}$ per cent: $27\frac{8}{10}$ per cent. in the manufacturing districts, and $4\frac{7}{10}$ in the agricultural.

The state of *house accommodation* is one element for measuring the prosperity of a people. In 1821, there were 1,142,602 inhabited houses; in 1831, 1,249,816, or an increase of $9\frac{6}{10}$ per cent., and in 1841, 1,328,839 or an increase of 7 per cent. "Nearly one-half of the families of the rural population, and somewhat more than one-third of the families of the civic population, are living in the lowest state, being possessed of accommodation equivalent to the cabin, consisting of but a single room. In the third class, but little removed in comfort, are nearly the same proportion, while of the other class (the house accommodation is divided into four classes) the number is extremely small."

*Occupation.*—Two-thirds of the male and more than one-third of the whole community are engaged in producing, preparing, and selling food. It is well observed that *juvenile labour* is in nowise confined to factory or mechanical employments. "It is a feature of the age, and pervades every class of society, arising doubtless, from the fact that greater numbers render both subsistence and destination more difficult than formerly."

*Education.*—The state of education in different parts of Ireland (as in different parts of England) exhibits great varieties, from the one extreme of 25·16 per cent. of population in Dublin five years old and upwards who can neither read nor write, to 79 per cent in Mayo. So, too, in Dublin, 25 per cent. of the population from 5 to 15 are attending schools, but only 8·3 in Mayo. At the period of returning the Census 1821, 394,814 children were in actual attendance at schools, and in 1841, 502,491.

*Marriages.*—Of the proportion of the married to the unmarried, taking all Ireland into account, we have the following statement. Of 100 males above 17 years of age in the rural districts there are 44 unmarried, 51 married, and 5 widowers: in the civic districts, 41 unmarried, 53 married, and 6 widowers. Of 100 females in the rural districts there are 38 unmarried, 50 married, and 12 widowed; and in the civic districts, 41 unmarried, 43 married, and 16 widowed. These and other tables, made for a population of more than eight million, confirm the results arrived at by Quetelet, in Belgium, that the *unmarried* are two-thirds of the community (about $\frac{2}{3}$ are under the age at which it is usually contracted), most numerous in rural districts, and among the male sex; and that the widows are twice as numerous as the widowers, and most so in civic districts.

As to the *Age* at which marriage is contracted, 7 per cent. of the males, and 19 per cent. females in rural districts; and 13 per cent. males and 21 per cent. females in civic districts, are married between the ages of 17 and 25. "The average age at which people marry is found to vary with the mortality of the district, and the short-lived populations of unhealthy towns are, in their early marriages, merely following the same law which operates upon the longer-lived rural population, who marry at a later period, each seeking the renewal of his race towards the middle period of his existence." By far the greatest number of marriages of both sexes occur between the years 26 and 35. Prior to the perusal of these statements we certainly believed that early marriage was far more prevalent in Ireland than it proves to be.

By 100 marriages in which neither parent had been married before, 228

children were produced; in which the husband had been married, before 164; in which the wife had been married before, 143; and in which both parents had been married before, 87.

As to the *Sex* of the child we have the following observations:—

"It is worthy of notice, that even within the period to which these marriages belong, (ten years) the proportion between the sexes of the children is on the whole consistent with the results given throughout Europe generally from marriages whose families are complete, the difference in the age of the parties producing the same effect at any given period of time that would be produced from the same parties by continuance of time. Thus the proportion of male to female children is about 106 to 100, and is somewhat higher in towns than in the country, though it differs considerably in different years. The children appear generally to follow the sex of the elder parent, but this result is not invariable. On the contrary, it will be seen that when the age of the father exceeds by as much as twenty years that of the mother, especially in second marriages, the female births preponderate; from which circumstance, as well as some others which the tables indicate, it would appear that difference of age has a different value at different periods of life; or, in other words, that the sum as well as the difference of the ages of the parents ought to form an element in a calculation of probabilities on the sex of children.

"It may also be worthy of remark that, if the children of first marriages during the first five years of these tables be totalled separately from those of the five last years, the male births will somewhat predominate in the latter; from which it perhaps may be inferred that the early births of any given marriage are likely to be male, and that after a few years females begin to predominate; which may also point to the conclusion just adverted to, viz. that difference of age diminishes in its effect as the age of both parties advance. If tables of this nature were regularly collected, interesting results of this class might safely be drawn from them. We content ourselves, however, with forming these tables rather as a groundwork for future comparison, than as yet furnishing sufficient data for statistic calculation. But, we may remark in passing, that it will be seen, by reference to the table of Births, which gives the births of the whole community during the last 10 years, that the proportion of males to females is only 104 to 100, instead of 106 to 100, as in these tables, which give only the births from marriages which have taken place since 1830: whence it would appear that the number of female children has been greater in the marriages which still continued productive though they had taken place before 1830."

*Deaths.*—The number of deaths registered as occurring in the 10 years 1831-41, amounting to 1,187,374, is of course a lower one than the number which really occurred: owing to the emigration and extinction of some families, and the forgetfulness of others who furnished the returns, which were alone obtained from the voluntary statements furnished by the families in existence on the night of the 6th June, 1841. The Commissioners believe the deficiency may be rated as high as one-fourth. But they add—

"On the whole, we think it will be admitted, that however defective these returns of deaths may be in absolute numbers, they possess an extraordinary interest as a classified and digested record of the *causes* of more than a million of deaths. On these subjects we refer to the Report of Surgeon Wilde, of whose services we have availed ourselves in their compilation, abstaining from any comment of our own on the prevailing sources of mortality and disease, from feeling that his own views and ideas should rest upon his own responsibility.

Surgeon Wilde has devoted much labour and ability to the subject, and as these returns show the distribution and prevalence of disease, they will not be without a peculiar utility at the present moment, while the consideration of that subject excites so much public attention."

When we state that all these deaths are classed and tabulated, so as to show in what proportions the various diseases which have produced them have prevailed in every country district and town of importance of Ireland, their commanding utility to the statesman and philanthropist must be apparent. Our object, however, is rather with some of the general results than with the examination of the relative condition of particular divisions of the country; and we will content ourselves with the following statement upon this point, viz. that for all Ireland the average age at death for *males*, has proved to be 29·6 in rural and 24·1 in civic districts; and for *females*, 28·9 in rural and 24·3 in civic districts: but for the separate provinces it is as follows:—*Leinster*, Males 32 rural, 25 civic: females 31·5 rural, 25·4 civic. In *Ulster*, males 31·8 rural, 23·8 civic: females 32 rural and 23·6 civic. In *Munster*, males 28·2 rural, 23·6 civic: females 27 rural, 23·7 civic. In *Connaught*, males 26·1 rural, 22·6 civic; females 24·3 rural, 22·4 civic.

"The remarkable difference in the duration of life in Leinster and Ulster over Connaught and Munster, is too striking to be overlooked. The latter are the most exclusively agricultural, and from the analogy of Great Britain, should, on that account, seem likely to present the longest, rather than the shortest, average duration of existence. We fear, however, that the very low state, as to food and accommodation, of the rural population of these provinces, would be found, by a more searching inquiry and comparison, to place them in a sanatory point of view, more nearly equal with the crowded inhabitants of the western parts of England and Scotland, rather than the healthy rustics of the English and Scotch agricultural counties."

Mr. Wilde not only arranged this 1,187,374 deaths thus reported, according to their various causes, ages, and years of occurrence, but obtained additional returns from the various Hospitals, &c. a return of 25,378 deaths from cholera from the Board of Health, a return of deaths and executions occurring in jails, and returns of coroners' inquests and deaths in lunatic asylums. He thus left no source of information unapplied to: and followed in arranging the whole mass for the most part the nosological classification recommended by Mr. Farr,* as the simplest and most scientific yet proposed, and as offering facilities for the comparison of the diseases and mortality of the two countries. He comprises the whole, however, under 93 causes of death only, Mr. Farr enumerating 145.

The detail of these causes of death is preceded by an interesting disquisition upon the antiquity of the practice of medicine in Ireland. It would indeed be expected that the country which, when the rest of Europe was

---

* Those of our readers who do not possess Mr. Farr's pamphlet, detailing his Statistical Nosology, should apply for the same at the Registrar's Office, where they may obtain it gratuitously. It is of great importance that medical men should do all in their power to render the registration of deaths as accurate as possible, as regards nomenclature; for if this be left to the friends of the deceased, much of the value of the returns becomes diminished.

plunged in barbaric ignorance, cultivated learning, music, and other attainments of a politer age with earnestness and success, should excel in the simple medical knowledge of the period. The nation which could supply Charlemagne with his preceptor, and Alfred the Great with learned companions, would not be deficient in medical seers. But when our author traces the cultivation of what deserves to be styled medicine, to a period coeval with or anterior to the Christian Æra, we think that the national passion for antiquity, so conspicuous in various historical records, somewhat predominates. None of the writers upon medicine, medieval or modern, furnish an enumeration of the diseases of the country, and no General Bill of Mortality has ever been drawn up before the present, and even Bills for the City of Dublin, if ever they were kept with regularity, have long ceased to be so.

In the First Grand Class, viz. the *Epidemic, Endemic, and Contagious Diseases,* the total number of deaths amounted to 381,249 ; as 100 males to 92·93 females. A few condensed observations upon each of the sixteen diseases constituting these, may be interesting. We will enumerate them in their order of relative frequency.

1. *Fever.*—From this, the Plague of Ireland, (it is doubtful whether the true plague ever visited Ireland,) the Census-returns furnish 112,072 deaths: 100 males to 86·14 females, and 1 death in every 10·59 of the mortality from all causes, and 1 in 3·4 of the deaths of the total epidemic class of diseases. The spotted fever or true *Typhus Hibernicus* is recorded in the early Irish MSS. and existed in the island probably long before any records of its appearance. Although probably always endemic, the first authentic account of its appearing epidemically occurs as late as 1708, since when numerous similar visitations have been recorded. The present returns however prove that the statements of mortality, in some of these roughly put down at 60,000 or 80,000, must have been exaggerated. Drs. Barker and Cheyne calculated the number of persons attacked by the celebrated epidemic of 1817-19 at a million and a half. It spared neither rank or locality, and 100,737 persons were admitted into the hospitals, of whom 4,349 died. These writers are confirmed, by the present Census, in their opinion of the greater prevalence of the disease among males. This was the last pestilential epidemic in Ireland prior to the appearance of cholera in 1832. It is singular that fever has raged nearly decennially.

2. *Small-pox.*—58,006 deaths, 100 males to 96·45 females. Proportion to general mortality—1 in 20·46, and 1 in 6·57 to epidemic disease. The disease especially prevailed in 1837-8. Although constituting so large a share of the mortality, this disease, notwithstanding the rapid progress of population, is but one-half so prevalent in the City of Dublin as it was a century ago. Vaccination was long neglected in Ireland, and even now, in spite of its illegality, people pass from village to village for the express purpose of inoculating children. Two or more epidemics have usually prevailed simultaneously in Ireland, and thus when fever especially prevailed in 1740 and 1817, small-pox likewise raged epidemically and with great fatality. The public hospital returns only give 129 deaths in the ten years.

3. *Cholera.*—"The total deaths from this disease are 50,769: but as its duration was limited, being epidemic at most for only three years, 1832-4, it is only in comparison with the general deaths of these years that any fair or just proportion can be made. Upon this period its comparison with the deaths from all causes is 1 in 7·36, and compared with those of the epidemic class, 1 in 2·77. After the year 1834, when 4,419 deaths are returned, the mortality from this cause fell rapidly till the years 1837-8, when it rose again to 968 and 1,222." No age from 1 to 70 was spared by this disease, but it prevailed most from 35 to 40, and from 45 to 50. In the Dublin Cholera Hospital Records the sexes were 100 males to 137·34 females. Upon its first outbreak in 1832 the cholera prevailed most in the towns of the civic districts; and in 1833 it had spread through the country at large, and prevailed most in the rural districts.

4. *Croup.*—42,705 deaths, 100 males to 82·89 females. The males preponderate over the females at every age registered. It prevailed in the rural, compared to civic districts, as 40 to 27; and in comparison with the general mortality 1 in 27·8, with other epidemics 1 in 8·92. As the great numbers above-mentioned were furnished by persons not conversant with nice distinctions, several cases of other diseases may have been included.

5. *Hooping-Cough.*—36,298 deaths (21,325 within the first year), the proportion of the sexes being reverse to that which prevails in croup, viz. 100 males to 115·43 females. In comparison with other mortality, 1 in 32·71, and with epidemics 1 in 10·5.

6. *Measles.*—Deaths 30,739. Males 100 to 96·12 females. Compared with all diseases, mortality 1 in 38·62, with epidemic 1 in 12·4. It was most fatal from birth to the end of the first year; and more than a third of all the deaths occurred in the civic districts. The Irish call it "the boiling disease," and it is first noticed in Irish records of the 16th century.

7. *Pemphigus.*—17,799 deaths, as 100 males to 78·93 females: 1 in 66·72 deaths, and epidemics 1 in 21·42. This, the *pemphigus gangrænosus*, so named by Dr. Whiteley Stokes, has long proved a most destructive disease in Ireland, and is known to the people by the names of "Mortifying Hive," "Burnt Holes," "Eating Disorder," &c. Dr. Speer observed it was confined to children of three months old, and from that to five years, but it has been observed near Dublin in children nine years old. The children of the poor, and those of them who live in damp situations, are especially liable, although the finest of the family is usually attacked. The disease as well as its modification Pompholyx, has been seen in adults, and four cases are in the present hospital returns. It prevails especially in rural districts, and in particular counties. In Ulster it was in proportion to other deaths 1 in 17·8, in Connaught 1 in 250·27.

8. *Diarrhœa and Dysentery.*—10,744 deaths, 100 males to 68·42 females, 1 in 110·51 general deaths, and 1 in 35·48 epidemic. This is a return that can be little depended upon, seeing the diarrhœa must frequently have been the mere symptom of various fatal diseases, rather than of dysentery, properly so-called. Dysentery seems in ancient times to have been endemic in various parts of Ireland, and broke out in occasional violent epidemics, especially among the soldiery. The last period when it was generally and fatally prevalent was 1818; but it has decreased much of late years.

9. *Influenza.*—Deaths 10,575, as 100 males to 84·65 females: being 1 in 112·28 general, and 1 in 36·05 epidemic deaths. Accounts exist of the epidemic prevalence of this disease from time to time from the beginning of the 17th century; but no very violent outbreak occurred until 1833-4, and again a very fatal one in 1836-7; during which last, Dr. Graves calculates the direct mortality from influenza alone in Dublin amounted to 4000. But Mr. Wilde considers this a great over-statement.

10. *Scarlatina.*—Deaths, 7,886; males 100 to 95·97 females; 1 in 150·56 of the total recorded mortality, and 1 in 48·34 of the epidemical. Like cholera, it was especially fatal in towns and crowded districts; and in this respect it is thus compared with other epidemics, 1 death in 24·63 in civic districts, and 1 in 65·07 in rural districts. It is not so destructive as other epidemics during the first year; but $\frac{10}{11}$ of the total died between the 1st year and the 10th.

11. *Thrush.*—Deaths 1480. Males 100 to 116·06 females. 1 Death in 802·27 of the entire mortality: of these 1147 occurred in the first year, and 300 from the 1st to the 5th.

12. *Erysipelas.*—Deaths 1232. Males 100 to 73·52 females. 1 Death in 963·77 from all causes, and 1 in 309·45 from epidemic. Most of the cases recorded were doubtless sporadic, the epidemic appearance of the disease being confined to hospitals. The disease prevailed most in the civic districts.

13. *Ague.*—Deaths 518. Males 100 to females 44·28. This disease is of comparative rare occurrence, and in course of great diminution in Ireland. By some it has been considered entirely an imported disease, and the greater frequency of its occurrence and mortality in the West of Ireland, the chief locality of the annual emigration to and from England, gives support to the opinion. Ague was a cause of death in frequency to the general mortality in the following proportions: in Leinster 1 in 2,548·77: in Munster 1 in 2,115·73: in Ulster 1 in 3,005·61: and in Connaught 1 in 1,696·04.

14. *Syphilis.*—Deaths 384. Males 171, females 213. In reference to the sex, the disproportion has arisen from the women dying in hospitals, and the men in many cases returning home, and their deaths being registered as from other causes. Of course the returns for a disease of this nature are not to be relied upon.

15. *Hydrophobia.*—There are 31 deaths registered as occurring from this cause—24 males and 7 females. Three of these, reported to have occurred during the first year, may be doubted.

16. *The Glanders* destroyed 10 males and 1 female.

SPORADIC DISEASES.—We can only notice some of the most important of these.

1. *The Nervous System.*—Total deaths 104,063. Males 100 to females 73·87. These deaths, compared with the general mortality, amount to 1 in 11·41.

*Hydrocephalus.*—Deaths 8,997. Males 100 to 78·93 females. During the first year the deaths amounted to 3002; from the first to the end of the fifth, to 3189 ; from the fifth to the tenth inclusive, to 1817 ; and from that age upwards, to 976 ; in this latter number must have been included a large proportion of diseases of the brain not generally considered as examples of hydrocephalus.

*Apoplexy.*—Deaths 11,185. Males 100 to 56·82 females. Deaths in hospitals 139 males and 52 females. The rural districts have afforded the chief mortality.

*Convulsions.*—Deaths 66,870. Males 100 to 76·78 females. Proportion to total deaths 1 in 17·75. This is the prominent symptom, and hence the term popularly assigned to a vast number of infantile diseases. During the first twelve months 54,822 are registered as from this cause: from the first to the end of the fifth year 10,610 ; and from the fifth to the fifteenth year 1433. After this age convulsive diseases are registered as epilepsy.

*Lock-jaw.*—Deaths 238. Males 172, females 66. There are registered 40 cases as occurring during the first month—doubtless instances of *trismus nascentium*, which Dr. Collins states occurred in 37 out of 274 deaths of children in the Dublin Lying-in Hospital ; and Dr. Joseph Clarke records that in his time $\frac{19}{20}$ of the great mortality which then prevailed in that institution arose from this cause.

*Epilepsy.*—Deaths 1030. Males 100 to 68·57 females. The males predominate at every age except from 5 to 10 and 15 to 20, when females are most numerous. The disease proved most fatal at the ages from 10 to 15 and 20 to 40.

2. *Respiratory and Circulatory Organs.*—These amount to 178,919 deaths in the proportion of 100 males to 101·72 females. Compared to the total deaths they are 1 in 6·63.

*Consumption.*—This is by far the most fatal of the diseases of Ireland, exceeding even fever by 23,518 deaths. The numbers are 135,590. Males 100 to 113·07 females. To deaths from all causes the proportion is 1 in 8·75, and to those of other affections of the respiratory and circulatory organs 1 in 1·31 : to epidemics in general as 100 to 281·17 : to fever alone as 100 to 82·65. The proportion in the rural compared to the civic districts is 100 to 25·71. The most fatal age was from 15 to 30th year and then from the 30th to the 50th.

*Inflammation of the Lungs.*—Deaths 27,684. Males 100 to females 63·11. The most fatal period was from the 45th to the 60th year. It is obvious that the returns concerning this disease, and many others of the chest, as bronchitis, pleurisy, hæmoptysis, diseased heart, &c. &c. must be very imperfect, derived as they are from popular sources alone.

*Asthma.*—Deaths 10,594. Males 100 to 102·98. Compared with total deaths 1 in 112·07. Fatal period especially from 45 to 75. Here many diseases must of course become confounded under one name.

3. *Digestive Organs.*—These deaths amount to 123,828. Males 100 to females 90·98. Compared to all others they are 1 in 9·58.

*Dentition.*—Deaths 3,303. Males 100 to 87·67 females. More than 2000 occurred during the first year, and several deaths entered as convulsions were due to this cause.

*Dropsy.*—(embracing anasarca as well as ascites) deaths 23,332. Males 100 to 87·67 females. This disease ranks thirteenth in frequency of all registered, and, next to consumption and fever, in the frequency of its occurrence in public hospitals, &c. Acute dropsy is not an uncommon affection of childhood in Ireland: thus, 532 deaths occurred during the first twelvemonth, and 4,442 between the ages of 1 and 15.

*Marasmus.*—Deaths 68,650. Males 100 to 105·81 females. Next to convulsions it is the most destructive affection of extreme youth; but differs from that disease in the fact of the female exceeding the male deaths at all ages after the first year. During the first five years it is 1 to 0·86 compared with small-pox: 1 to 0·43 with measles: 1 to 0·09 scarlatina; 1 to 0·59 hooping cough; 1 to 0·7 croup: 1 to 1·16 convulsions; and of the general mortality to the same period 1 in 7·67. This disease is a favourite one among the lower orders for the exhibition of several superstitious practices for its cure. Believing it to originate in the malevolent power of the fairy tribe, they employ various means for circumventing or propitiating this minute but mischievous race.

*Urinary Organs.*—The total deaths from diseases of these, amount to 3,137. Males 100 to 14·07 females.

*Childbed,* or its immediate consequences, puerperal fever, flooding and inflammation, proved fatal to 13,903—383 cases of which occurred in the hospitals, &c.; 1562 in the civic districts (or 1 in 149·91 of the entire deaths of the towns of or above 2000 inhabitants); and 11,958 in rural districts, or 1 in 76·23 of all the deaths of the minor towns and the open country. Of these deaths, 9 occurred at or under 15 years of age: 563 from 15 to 20: 6603 from 20 to 30; 5567 from 30 to 40: 1084 from 40 to 50: 42 deaths from 50 to 55; and 10 deaths from 55 to 60—1 death occurring at 66, the age of the husband then being 72 and the date of the contraction of the marriage 1780.

*Rheumatism.*—Deaths 3511. Males 100 to 80·97 females. It seems especially prevalent and fatal in rural districts. It is termed in the vernacular "splitting of the bones;" and "sweating houses, a rude species of Russian bath, are still in vogue for the cure of rheumatic affections, particularly in the western parts of Ireland." The great majority of deaths occurred between 45 and 80.

*Scrofula.*—"Were the history and symptoms of at least one half of the deaths from the sporadic diseases enumerated in the 671,960 deaths from these causes accurately known, it would perhaps fall short of the number of deaths that might be traced directly or indirectly to this taint; for tubercle, in one form or other, is one of the most frequent causes of death in this country. The deaths specified in the Census returns, "scrofula," "the evil," the "King's" or "running evil" amount to 3149, as 100 males to 64·01 females.

VIOLENT AND SUDDEN DEATHS.—These amounted in all to 44,445, as 100 males to 49·3 females; 1 in 25·92 occurring in rural districts, and 1 in 29·54 in civic districts. Compared to epidemics they are 1 to 8·57, and to sporadic diseases 1 to 17·13. *Burns and Scalds* destroyed 4349 persons. To the end of the fifth year the sexes are nearly equal; but from that age upwards the female deaths predominate. The great majority of deaths occurred from the twelfth month to the end of the twelfth year. Deaths from *drowning* chiefly accidental, have amounted to 7072. Males

100 to 29·45 females. Deaths from *Intemperance* amounted to 1239, or 1043 males and 196 females. The intemperate abuse of spirituous liquors existed in Ireland, as in England, especially about 1724; and, some time after then, the places for selling liquors in Dublin alone amounted to 3500. From that time the habit of drinking continued to prevail in great excess in Ireland, and was followed by a black train of consequences, in the shape of deaths, diseases, and insanity. Death from this cause increased rapidly from 1831 to 1839. Happily a new order of things commenced, and has already borne its fruits. The places in Dublin for retailing liquors in 1841 only amounted to 1098, an astonishing decrease when we consider that 3500 existed a century ago with a so much smaller population. Much however remains to be done; for, 13,204 persons (7,544 males and 5,660 females) were apprehended for being drunk in the streets of Dublin during 1841. A less quantity of ardent spirits is taken by the lower orders in Ireland than by the inhabitants of the large manufacturing towns in England; but, as formerly observed by Dr. Speer and now reiterated by Mr. Wilde, the injurious effects of these pernicious compounds are much increased by the former taking them in the morning upon empty stomachs, while the latter accompany their use with a considerable portion of solid nourishing food.

*Executions.*—There were 197 persons (188 males and 9 females) executed during the ten years: but a comparison of the first five years with the latter five years shows that the annual average was 31 in the former and but 8 in the latter. The greatest number of executions occurred between the ages of 20 and 25.

*Accidental Poisoning.*—Deaths 139. Males 74, females 65. Proportion to violent deaths 1 in 319·74: and to general mortality 1 in 8,542·25.

"The *total deaths* from all sources for the entire kingdom registered from the Census returns, and which purport to be the deaths of the families from whom these returns were received, during the ten years ending 6th June 1841, amount to 1,187,374 in the proportion of 100 males to 91·24 females: of which number 911,619 occurred in the open country, including the minor towns and villages, the sexes being 100 males to 91·04 females—234,173 deaths took place in the civic district, as 100 males to 94·05 females, and 41,582 deaths in the different hospitals and sanitary institutions, in the ratio of 100 males to 80·67 females. But as 39,950 of these deaths occurred in those hospitals which were situated in the towns that form the civic districts, it increases the mortality of these places to 274,123, or 1 in 4·14; and the remainder added to the rural districts in which the hospitals where they happened are located, raises its mortality to 913,251, or 1 in 7·7."

Upon the *proportionate mortality* we have the following observations.

"This mortality, which according to the first series of calculations, is nearly 1 in 55, and by the second, or that in the year 1840, is 1 in 57.19, may appear at first sight too small. It is, nevertheless, I am of opinion, but little short of the truth, especially when the circumstances of Ireland are considered in comparison with those of England, where, in the period from 1821 to 1831, the average annual mortality was but one in 51; and by the present more accurate system of registration it varies from 1 in 47 in 1839 to 1 in 44 in 1841, and the average of the entire period during which the Registration Act has been in force, is as low as 1 in 45. In England, the Bills of Mortality for many years, and the lately published Sanitary Report upon the condition of the working classes in particular, show us that the ratio of deaths to the living varies from 1 to 64 in agricultural districts and the open country, as in Herefordshire, to 1 in 28.33 in the densely populated manufacturing towns, such as Manchester, &c. To establish, then, a fair analogy between the mortality of the two countries, taking England as the average, the proportion of Ireland which is analogous to, and its inhabitants similarly situated with, those of the working classes in Herefordshire, Dorset, or Cornwall, the healthiest parts of England, on the one hand—and the proportion of this country, or its inhabitants similarly situated with those of Leeds, Manchester, or Liverpool, the unhealthy parts of England, on the other, must be all taken into account: and thus comparisons may be formed between the relative mortality of the two countries. The late Sanitary Report exhibits conditions of the population in the mining and manufacturing districts of England, such as are not to be found in this country; the infantile mortality in the most unhealthy parts of Ireland, falls far short of that in some of the larger towns in England; and, lastly, the proportion of sudden or violent deaths is 745 in every 100,000 of the population in the latter, and but 61·22 in 100,000 on an average of five years in the former country. From an examination of the entire records of death, disease, and longevity, in this country, I am led to believe that, were an accurate registration effected, the *proportionate* annual mortality would not be found to exceed 1 in 52 or 1 in 53."

Of course Mr. Wilde's calculations can, in the absence of an actual registration of deaths, only be approximative; besides, the deficiencies in the return of the number of deaths already noticed, scarcely any still-born deaths were returned, which, at 4 per cent. for the 10 years would amount to 90,000; and the instances were very numerous where even the hospitals could furnish no return of their dead. The Reporter has ably contended against these various difficulties, and the conclusions he has arrived at will probably be confirmed when a proper system of registration is established.

*Coroner's Inquests.*—A return of 20,334 deaths upon which coroner's inquests were held was obtained. Of these there were 3,494 *homicides* of several varieties, all of which exhibit an annual average decrease of 74·6 during the last five years. *Suicides* 755, which have, during the last five years, increased 13 on the annual average. Nearly $\frac{1}{3}$ of these deaths were caused by hanging; about $\frac{1}{5}$ by drowning; less than $\frac{1}{10}$ by stabbing: somewhat more than $\frac{1}{12}$ by poison; more than $\frac{1}{17}$ by shooting; and scarcely $\frac{1}{37}$ by suffocation. There were 8072 *accidental deaths*, of which drowning gives 2410, burning 532, cold 495, suffocation 369, poison 68, lightning 38, unspecified 2811. In deaths caused by fire, females predominated, in all others, the males. Deaths from *immoderate use of ardent spirits,* amounted to 1129, *i. e.* 950 males and 179 females. In 12,762 inquests, medical evidence was had recourse to. "In 315 inquests held upon new-born infants, the Coroner's juries determined, without the examination of medical evidence, that 99 were still-born; while in 79 instances of accidental gun-shot wounds, medical evidence was had recourse to in 60 cases." A truly Hibernian mode of procedure.

*Insanity and Idiotcy.*—The returns embrace the deaths of such as were received into the different asylums (public and private) and jails during the 10 years: and of the living under confinement on 6th June, 1841. Of the 2,778 deaths returned (1528 males and 1250 females) the immediate cause of death is stated only in 2,161.

"The examination of this table shows us not only the vast preponderance of deaths from diseases of the nervous system, the most usual causes of insanity, idiotcy, and epilepsy, amounting to 791, but also of those affections that in an especial manner mark the *scrofulous* nature of lunacy. Thus the deaths from consumption, including, in all probability, marasmus and maniacal decay, as well as phthisis, number 601 alone: dropsy, symptomatic, in all probability, of tubercular disease of the abdominal viscera 37: disease of the liver 9; and scrofula, so termed from some manifest exhibition of the affection, 32; in all, 1,470, or somewhat more than one-half of the entire deaths."

The proportion of males to females in the total deaths was 100 to 81. The greatest mortality prevailed between 20 and 50; and the average duration of life was 35 to 40 males, 40 to 45 females. Comparing the deaths and receptions in ten district asylums, the average mortality was, in the ten years, 20·45 males, 17·37 females, 19·02 on both. In the five public asylums of Cork, it was 26·18 males, 22.2 females, 24·22 on the whole. The Friends at Donnybrook gave but 5·18 per cent. In eight private asylums, the mortality averaged 12·02.

The lunatics under treatment in the different asylums and jails, on 6th June, 1841, amounted to 3,382 persons—of whom 333 were idiots, and 124 epileptics. The mean period of life was from 30 to 40. The educated persons were 2,088, the uneducated 1,294. Males 1,209 educated to 527 uneducated. Females 879 to 767. There were married 411 males to 504 females, and 1,325 males to 1,142 females unmarried.

*Hospitals and Sanitary Institutions.*—The Infirmaries or County Hospitals amount to 35, and the General Hospitals, situated chiefly in the cities and large towns, to 30. The following is the proportionate mortality from the various classes of disease. Epidemic, Contagious, and Endemic, 2,734, of which 2,526 were cases of fever. Diseases of Nervous System, 972. Respiratory and Circulatory Organs, 2,366. Digestive Organs, 2,041. Urinary Organs, 208. Locomotive Organs, 413. Tegumentary System, 325. Uncertain Seat, 1,776. It is almost incredible that 6,049 instances, or nearly one in every three cases occurring in these public institutions are returned as the cause of death not being known; nay, more inexcusable still, the records do not specify the *age* in 8,764 instances!

The total amount of deaths in 86 Fever Hospitals from which returns were received, amounted to 15,988. Males 100 to 94·5 females, "but, from the defective state of Hospital Registries in Ireland, this falls far short of the actual number."

*Lying-in Hospitals.*—Eleven printed returns of deaths of 394 mothers, and 2,258 children, but in 2,249 the cause of death of the latter not returned, many, however, being still-born. The ratio of still-born was 6·87 per cent. This is not the average ratio throughout the country, which is not more than four or five per cent., but the maximum occurring in crowded cities and public institutions.

The number of patients in the Hospitals and Lunatic Asylums, 6th June 1841, amounted to 8,652, as 100 males to 97·71 females; their proportion to the entire population of Ireland being one in 944·88.

*Sanitary Report on the City of Dublin.*—This contains a map of Dublin, coloured and shaded in different manners so as to show at one view its healthy and unhealthy localities; and must be of great utility to all engaged in investigating the condition of that city, Mr. Wilde has classified the whole of the streets and distinguished each class by its characteristic colour, and the various intervening spaces by different shading. He has examined the total mortality of Dublin, for the 10 years, amounting to 66,722, and distributed it over its approximate localities, not for the purpose of arriving at the actual mortality, which the defectiveness of the returns prevented, but at the relative healthiness of the various localities. In the most fashionable and healthy parts of Dublin, the deaths were one in 122·2, while in the lowest and most wretched parts they varied from one in 37·8 to one in 43·8.

Among other interesting investigations contained in this department of his Report, Mr. Wilde enters into a comparison of the prevalence of mortality, as ascertained by cemetery returns and meteorological observations, in certain seasons of the year.

"From an examination of this table, which is the average of two years, we learn that the months of May, June, and July, are the healthiest, and next those of August, September, and October: but, in fact, those two periods of Summer and Autumn are so analogous, that, as far as health and mortality is affected, the year divides itself into two equal periods by the months of May, and November, the mortality of the first being to the second as 100 to 137.16. December, January, and March are the trying seasons; and the deaths of children under five years of age predominate in November, December, January and March. We also learn, from his accurate registration, that the sexes vary with the seasons, the females being in proportion to the males least in Autumn and Winter, and greatest in Spring."

We repeat, that this Report is a valuable contribution to the Vital Statistics of the Sister Kingdom; and we hope that the Legislature will place, ere long, in Mr. Wilde's hands, by means of a General Registration Act, the power of corroborating or correcting its various inferences.

